# Sensor Data Prediction in Missile Flight Tests

**DOI:** 10.3390/s22239410

**Published:** 2022-12-02

**Authors:** Sang-Gyu Ryu, Jae Jin Jeong, David Hyunchul Shim

**Affiliations:** 1 Department of Electrical Engineering, Korea Advanced Institute of Science and Technology (KAIST), 291, Daehak-ro, Yuseong-gu, Daejeon 34141, Republic of Korea; 2 The 1st R&D Institute, Agency for Defense Development (ADD), 160, Bugyuseong-daero 488 Beon-gil, Yuseong-gu, Daejeon 34060, Republic of Korea; 3 Department of Electronic Engineering, Kumoh National Institute of Technology, 61, Daehak-ro, Gumi-si 39177, Republic of Korea

**Keywords:** sensor data prediction, time series prediction, deep learning, long short-term memory, recurrent neural network, missing data imputation, wavelet reconstruction

## Abstract

Sensor data from missile flights are highly valuable, as a test requires considerable resources, but some sensors may be detached or fail to collect data. Remotely acquired missile sensor data are incomplete, and the correlations between the missile data are complex, which results in the prediction of sensor data being difficult. This article proposes a deep learning-based prediction network combined with the wavelet analysis method. The proposed network includes an imputer network and a prediction network. In the imputer network, the data are decomposed using wavelet transform, and the generative adversarial networks assist the decomposed data in reproducing the detailed information. The prediction network consists of long short-term memory with an attention and dilation network for accurate prediction. In the test, the actual sensor data from missile flights were used. For the performance evaluation, the test was conducted from the data with no missing values to the data with five different missing rates. The test results showed that the proposed system predicts the missile sensor most accurately in all cases. In the frequency analysis, the proposed system has similar frequency responses to the actual sensors and showed that the proposed system accurately predicted the sensors in both tendency and frequency aspects.

## 1. Introduction

Prediction of time series sensor data is becoming increasingly important as sensors proliferate and are used in almost all applications. Predicting sensor data can reduce costs and make up for compromised or faulty sensors, so the needs are increasing in many areas. Examples are the prediction of temperature in the industry [1], health-related applications [2], and the pandemic of COVID-19 [3].

In missile development, sensor data obtained from flight tests are valuable, as a test requires many resources and members of the workforce. Further, the data can also be used in the analysis for troubleshooting, performance improvement, and missile design. In a flight test, unexpected situations may occur, and the measured sensor data may be compromised. At times, sensors are removed after verification of missile design, but sensor data may be needed to solve problems. The prediction algorithm can reproduce corrupted or removed sensor data in these cases.

For the prediction of sensor data, model-based methods can reproduce the data with high accuracy and high sensitivity when the environments are limited (in the laboratory) and when additional human endeavors or apparatus exist in advance [4]. However, the real-world environment varies, and a complex model should be prepared by trial and error to reproduce it. In the case of using multiple sensors, as in missile tests, there must be many tests and human resources to get accurate models for multiple sensors. In preparation for the case of sensor data loss, it is not cost-effective to produce all the models, and the prediction of missile sensor data should be achieved by utilizing the previous data, which is the data-driven method.

For these reasons, data-driven methods utilize statistical techniques or deep learning-based achievements in predicting various sensor data. Traditional data-driven methods are the auto regressive moving average model (ARMA) and the auto regressive integrated moving average model (ARIMA). These models, however, have difficulty achieving high performance in the prediction of real-world sensors due to the complexity, irregularity, randomness, and nonlinearity of the data [5]. For better performance with these methods, the data need expert adjustments (e.g., deleting errors, normalizing the data, adding/mixing/deleting some data vectors, and interpolations), and the results depend on the expert’s ability.

These problems can be solved using a deep learning approach. In the past decade, deep learning-based prediction algorithms with various network structures have been studied, and the data can be predicted more accurately with a deep learning network. With a deep learning network, data can be normalized and adjusted with little human intervention (Table 1). Based on these advantages and characteristics of the deep learning approach, we designed an effective deep learning-based network to predict the sensor data in missile flight tests.

To analyze data characteristics, many decomposition methods were developed to extract various components (e.g., seasonal, trend, and abrupt components) from the non-stationary time series for improved predictability and interpretability [6]. Among the methods, wavelet transform has become a widely used tool for signal analysis as a temporal-frequency representor. We combined wavelet transform, the powerful data analysis method, with a deep learning network to assist in the prediction of sensor data. Related work and details of the implementation follow.

## 2. Related Work

In our missile flight data, missing values exist due to loss of communication or corruption. Data imputation algorithms can help predict the output sensor data by making up for the missing values. In Section 2.1, previous work on data imputation algorithms is introduced. In Section 2.2, wavelet decomposition and reconstruction for data analysis are described, and generative adversarial network, which is an additional helper for an imputer network, is explained in Section 2.3. In Section 2.4, long short-term memory for the prediction network is explained.

### 2.1. Data Imputation

Imputation is widely used, as missing values can significantly affect performance [7], so missing filling algorithms have been introduced, e.g., k-nearest neighbor (kNN), tree-based, ensemble methods (bagging and boosting algorithm), and deep learning-based methods.

The kNN-based method imputes the missing data with the closest values or classes by computing the k number of the nearest normal data with the specific distance metric. In applications of kNN-based imputation, missing species-level forest biomass data are imputed [8]. The authors tested six distance metrics and fifteen k values to find optimal parameters in imputing biomass. The kNN-based methods are simple, but the performance varies depending on the parameters (distance metric, k value), and the proper parameters are needed to achieve high performance.

Tree-based methods were also used in imputation. Single tree-based algorithms have trouble with generalization and overfitting, so ensemble algorithms such as bagging and boosting have been introduced, resulting in better performance [9]. Among the bagging algorithms, random forest (RF) randomly selects features, makes a decision tree model, and iterates it to make multiple decision trees. The resultant decision trees are aggregated in the last step, and an improved tree-based imputation model is generated.

Boosting algorithms train and combine the learners by considering the importance of the data. In boosting algorithms, adaptive boosting (AdaBoost) arranges multiple learners sequentially by calculating the importance of learners through the Gini impurity measure. Each learner only considers a different single feature or combination of features in the data and is trained iteratively. In training, the “weight of the data observations” (concept of boosting) is adjusted through the error measure of the result from the previous iteration. The higher the weight of data observations, the larger the data are reflected in training.

Gradient boosting machine (GBM) trains the learners to minimize the difference (loss) between the true value and the output of learners. It weights the observation of the data by calculating the gradient of the loss. Generally, it updates the learners to the negative direction of the gradient as the loss metric is a mean squared error, but the loss metric can vary. In updating, the concept of learning rate is applied to prevent overfitting; however, the algorithm can still be overfitted.

Extreme gradient boosting (XGB) adds more complicated concepts to prevent overfitting, and a regularization term is one example. The regularization term considers the number of each tree node and node score, so it makes the tree not have too large a number of tree nodes and node scores by adding it to the loss function. Through multiple techniques, XGB has been successfully applied to the imputation of relatively large and complex datasets [10].

Various imputation algorithms have been developed and applied to many areas, but parameter tuning is essential for acceptable performance [11]. An example is that AdaBoost showed inferior performance to the simple kNN algorithm when the parameters were not optimized [12]. For stable performance, many researchers have studied and set the rules to select the effective parameters, but the algorithms still have many parameters to tune for high performance, such as the number of nodes, the number of trees, and the number of features. Further, thorough dependence consideration between the features is needed to handle a more complex dataset. The algorithms noted above consider the dependencies between the sensors less for computation simplicity, and deep learning-based imputation is a better approach to high dimensionality features, severe nonlinearities, and unconventional data [13].

For these reasons, there have been many deep learning-based approaches for imputation using various deep learning structures, such as convolution neural network (CNN) [14], recurrent neural network [15], and the generative adversarial network (GAN) [16]. In our problems, deep learning-based imputation is implemented, as the data are complex with high dimensional (the number of channels is over two hundred), nonlinear, and unconventional problems.

### 2.2. Wavelet Analysis

Wavelet transform (WT), as a time-frequency analysis method, has been successfully applied to an extraordinary range of time series applications. Wavelet filters exist over a finite time limit and are better suited for interpreting seasonal, trend, and abrupt components [6,17].

WT decomposes information into approximated and detailed parts. The approximated parts include low-frequency information, and the detailed parts include high frequency information. The approximated parts can be decomposed continuously, and the low-frequency information can be subdivided and analyzed more closely. Both the approximated and detailed parts are obtained using wavelet coefficients $a_{j,k}$, below.

(1) $$\psi_{j,k}\left(t\right)=2^{-j/2}\psi \left(2^{-j}t-k\right)$$

(2) $$x\left(t\right)=<a_{j,k},\psi_{j,k}\left(t\right)>=\sum_{j,k}a_{j,k}\cdot {\bar{\psi }}_{j,k}\left(t\right)$$

(3) $$a_{j,k}=<x\left(t\right),\psi_{j,k}\left(t\right)>=\int x\left(t\right)\cdot {\bar{\psi }}_{j,k}\left(t\right)dt$$

where ψ(t) is a kernel function, *j* is the dilation (number of scale), *k* is a translation, and x(t) is the original signal.

In WT, signals can be decomposed with the various kernel functions (e.g., Haar, Meyer, Morlet, Daubechies, Symlet, Coiflet). Each wavelet kernel has its characteristics, and some kernel functions can be more useful to the specific signal analysis. In electrocardiogram signal denoising, four different kinds of wavelet kernels (Haar, Daubechies, Symlet, and Coiflet) were used in wavelet decomposition, and the Symlet kernel showed the best performance [17].

Further, there are various wavelet analysis methods, such as continuous/discrete way, multi-resolution analysis, wavelet packet decomposition, wavelet reconstruction, maximal overlap discrete wavelet transform, and so on. This variety of kernel functions and types are the main advantage of WT [6].

**Figure 1:**
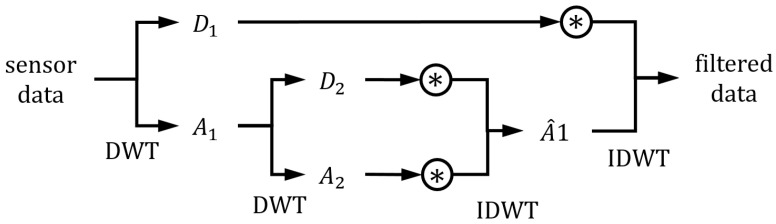
Structure of the wavelet reconstruction method. The convolution notation (asterisk with circle) represents the weighting process of the specific decomposed part.

In the various methods, the wavelet reconstruction method selectively analyzes multi-frequencies by repeatedly decomposing time series data. The approximated parts can be decomposed again by discrete wavelet transform (DWT), so data can be analyzed in multi-frequencies. The decomposed parts can be reconstructed by inverse discrete wavelet transform (IDWT), and specific parts can be weighted and inverse transformed for rejecting or weakening the specific parts. Figure 1 shows the wavelet reconstruction method with a wavelet level of two.

The wavelet analysis methods can improve performance when used with other methods. Time series data are forecasted more accurately when the wavelet transform was combined with ARMA and ARIMA compared to the results when only ARMA and ARIMA were used [18], and defects were effectively detected in steel images with the wavelet reconstruction method and particle swarm optimization [19].

The effectiveness of WT also has been combined with the powerful performance of the deep learning approach. Wavelet decomposition and CNNs were used to analyze returned waveform data for the prediction of water level [20]. In the image processing area, deep learning networks were applied to the wavelet transformed image for image inpainting [21], super-resolution [22], and brain tumor detection [23]. We also utilized the effectiveness of multi-frequency analysis in WT and the powerful performance of deep learning networks. The details of the implementation are stated in Section 3.2.

### 2.3. GAN

GAN was inspired by the game where a generator and a discriminator compete with each other. The generator imitates the real value to deceive the discriminator, and the discriminator detects the generator as fake [24]. The generator network (**G**) is trained to generate a value similar to the real value to fool the discriminator, and it is represented as minimizing log(1−D(G(z))). The discriminator network (**D**) is trained to discern the real value as real and the generated output as fake, and it is represented as maximizing log(D(x)). Simultaneous training of the generator and discriminator networks are represented below:

(4) $$\underset{G}{min}\underset{D}{max}\;V\left(D,G\right)=E_{x∼p_{data}\left(x\right)}\left[log\left(D\left(x\right)\right)\right]+E_{z∼p_{z}\left(z\right)}\left[log\left(1-D\left(G\left(z\right)\right)\right)\right]$$

where $p_{data}\left(x\right)$ is the real data distribution, $p_{z}\left(z\right)$ is the noise distribution, log(·) is cross-entropy, and D(·) and G(·) are the results of the discriminator and generator network, respectively.

For training GAN, each network is trained one by one. The generator network is fixed when the discriminator is training, and vice versa. In the process, the generator network can generate output very close to the real data due to the discriminator network. This characteristic makes the GAN useful in many applications, such as imputation [16], texture conversion [25], image inpainting [26], and fault diagnosis [27]. GAN tends to recover high frequency information as it focuses on detailed information to fool the discriminator [21,25], resulting in improved performance in many applications.

However, the generator and discriminator networks are trained separately, so learning stability and mode collapse are issues in GAN [28]. As the networks are trained one by one, GAN has inherent training loss instability, and mode collapse can occur when the generator focuses on the local distribution. The causes of these issues can vary, and there have been many kinds of research to handle these problems, from reengineering network architectures to new loss functions [29].

Among them, Wasserstein GAN with gradient penalty (WGAN-GP) improved the performance and stability by using Earth Mover’s distance and penalizing the norm of the discriminator gradient [30]. In this paper, we utilized WGAN-GP in recovering the detailed information by applying the adversarial loss to the decomposed data.

### 2.4. Long Short-Term Memory (LSTM)

Recurrent neural networks were introduced to consider time dependencies and relationships by connecting their units recurrently. The early stage of the recurrent network was recursive neural networks (RNNs). RNNs have been applied to time series applications [31], but they have problems with gradients exploding and vanishing when updating the networks. Gradient exploding can be solved by regularizing the weights. However, the gradient vanishing problem cannot be solved, as the gradient of the farther data becomes smaller in backpropagation. The limitation of these problems in learning long-term data motivated the different recurrent structures.

LSTM overcomes gradient vanishing and exploding problems by introducing the concept of forgetting [32]. The dependencies between the current input and the previous state are calculated as the weights through the sigmoid function. The weights, the dependency measure of how much the input is reflected in the cell state and affects the results, make differences in the learning process and result in forgetting less critical information and maintaining critical information.

**Figure 2:**
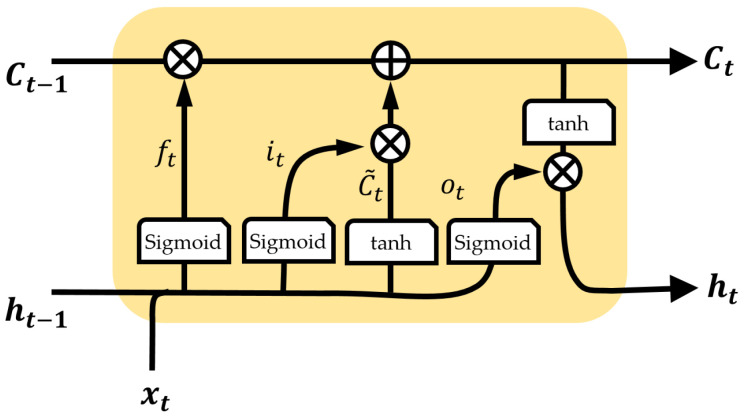
Structure of the fundamental LSTM network.

More specifically, an LSTM has internal parameters named cell state (*C*) and hidden state (*h*) as in Figure 2. The cell state preserves the previous information by weighting the previous cell state ($C_{t-1}$) with the forget weight ($f_{t}$) and reflects the input to the cell state through the newly estimated cell state (${\tilde{C}}_{t}$) from weighting it with the input weight ($i_{t}$). The cell state is updated not by multiplying but by weighting and adding, so the long-term information is preserved (multiplication in applying long-term information results in gradient vanishing in backpropagation).

The hidden state, which contains important information for predicting the current time, are filtered parameters from the cell state. The hidden state is updated by the tanh of the cell state ($C_{t}$) and weighting with output weight ($o_{t}$), and the output of the LSTM is the cell state and hidden state.

(5) $$f_{t}=\sigma \left(W_{f}\cdot \left[h_{t-1},x_{t}\right]+b_{f}\right)$$

(6) $$i_{t}=\sigma \left(W_{i}\cdot \left[h_{t-1},x_{t}\right]+b_{i}\right)$$

(7) $${\tilde{C}}_{t}=tanh\left(W_{C}\cdot \left[h_{t-1},x_{t}\right]+b_{C}\right)$$

(8) $$C_{t}=f_{t}⊙C_{t-1}+i_{t}⊙{\tilde{C}}_{t}$$

(9) $$o_{t}=\sigma \left(W_{o}\cdot \left[h_{t-1},x_{t}\right]+b_{o}\right)$$

(10) $$h_{t}=o_{t}⊙tanh\left(C_{t}\right)$$

where $x_{t}$ is the network input, $h_{t}$ is the hidden layer, *C* is the cell state with the subscript *t* and t−1 denoting time instant, ⊙ is element-wise multiplication, σ is the sigmoid function, and *W* and *b* are the weight and bias with the subscript f,i,C,o meaning the forget, input, cell, and output.

The property of long-term memory retention made LSTM to be applied in natural language translation [33], time series prediction [3,5,32], time series classification [34], and many other applications. The prediction performance of the simple recurrent network is inferior to the statistical ARIMA model [35,36], so most applications of LSTM was combined with other deep learning structures and techniques, e.g., fully-connected network, convolution neural network, autoencoder [32,33], squeeze-and-excitation block [34], and attention block [5].

Gated recurrent unit (GRU) is a simplified version of LSTM [37]. It is considered an efficient alternative to LSTM with a reduced number of parameters and a simpler structure [38], and it is unclear which network is better as it differs from application to application; LSTM is slightly superior to GRU in speech recognition [39]; GRU is superior to LSTM in control of a dynamic system [40]. Generally, however, LSTM is known as more fitted to complex data [41]. In our case, the data are more complex than the other applications, and we utilized the LSTM network with the attention mechanism and other deep learning techniques.

## 3. Missile System and the Proposed Network

### 3.1. Missile Data

**Figure 3:**
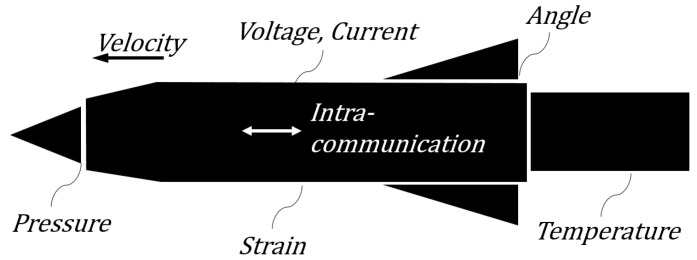
Types of data in a missile system.

In a missile system, various kinds of data are acquired by the remote acquisition system or the other relaying systems (Figure 3). Missile data consist of two parts: sensor data and intra-communication data. Sensor data are pressures, inner/outer temperatures of the missile, strain gauge readings from the missile body, acceleration, voltage, current sensors, etc. Intra-missile communication data include angles of fins, control-related data, missile attitude information, position-related information, the status of the equipment, etc. These sensor data and intra-communication data are used in controlling the missile guidance or analyzing the flight test result.

Each sensor or communication datum is digitized with a different sampling rate, and the time standard of the sensor or intra-communication data is different, so the data are out of sync. As a result, many data points are marked as not a number (NaN) or redundant values. Further, some data are missing when they are not received by the ground station. In Table 2, the time intervals of the data are not uniform, and some values are redundant or NaN.

**Figure 4:**
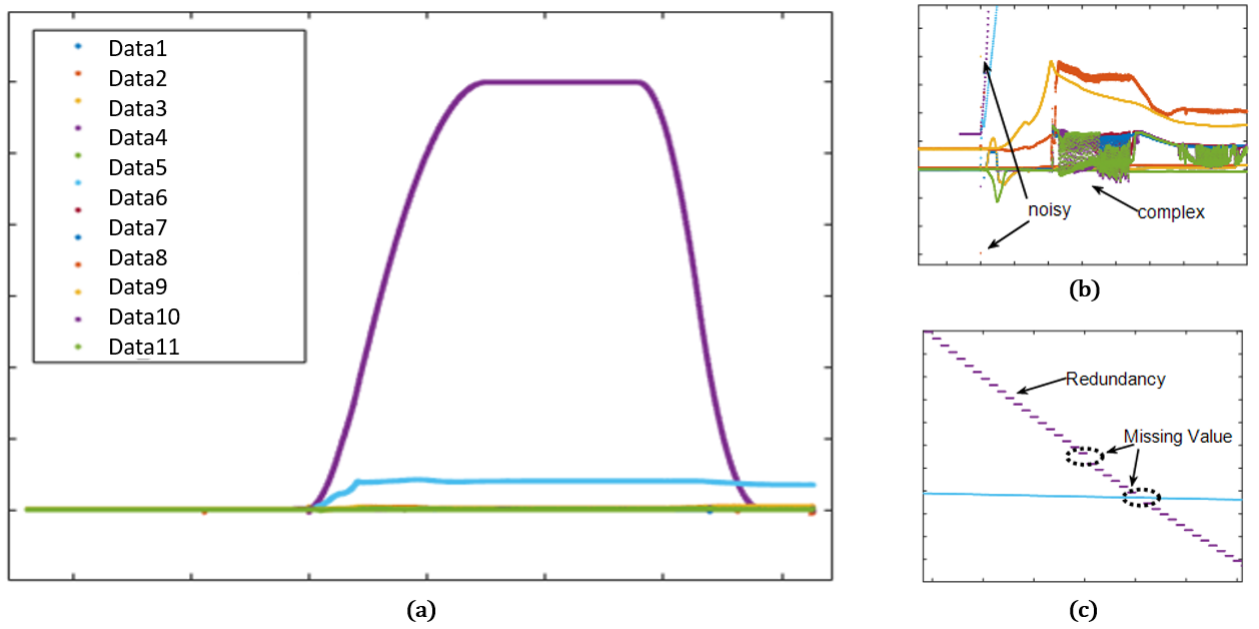
Missile data plot: (**a**) full view; (**b**) magnified plot with noisy and complex values; (**c**) magnified plot with redundant and missing values.

Figure 4 is the display of acquired missile flight data. The data seem simple and clear in the full view, but the magnified views (Figure 4b,c) show complex and noisy values with missing or redundant intervals. The missing data occur completely randomly and are sometimes continued for a random interval in case of communication loss. The complication and incompleteness in the missile data make it difficult to predict the sensor data accurately.

**Figure 5:**
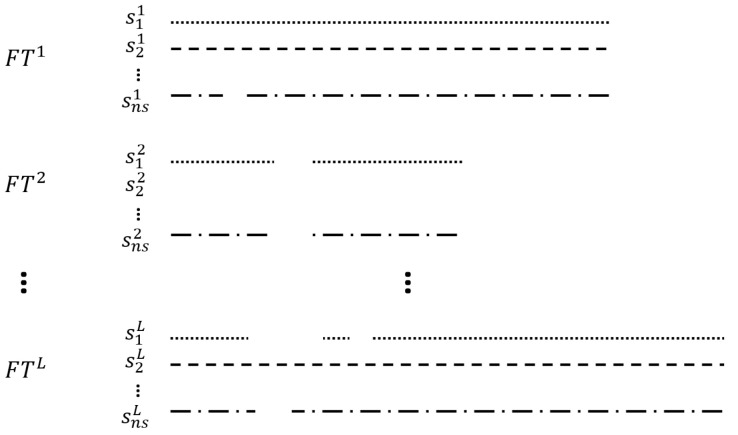
Examples of the missile dataset.

The proposed network predicts the missing sensor data utilizing the acquired dataset. For a clear explanation, examples of the missile dataset are shown in Figure 5. For the flight dataset (FT), the length of the lines in each FT shows the different test times, which means the test settings are different from test to test, as noted above. In sensor data (*s*), the superscript means flight dataset number with the maximum number of flight data *L*, and the subscript means sensor number with the maximum sensor number ns. The sampling rates can be different from sensor to sensor, and they are depicted as the different styles of the lines and dots, with blanks representing missing data. When all the sensor data are missing in a specific flight dataset ($s_{2,2}$), the proposed system can reproduce the missing sensor by using other flight datasets besides the $FT_{2}$.

### 3.2. Network Architecture

The sensor data from missile flight tests are complex, noisy, and have many redundant values, uncertain dependencies, and a large amount of data, as shown above. Although various deep learning and signal analysis methods have been implemented for prediction, there is a limit to predicting the sensor value due to the aforementioned characteristics. To accurately predict the sensor data, we combined various analysis methods and deep learning structures; wavelet is utilized for decomposing the complex sensor data into multiple characteristics; GAN helps to handle missing or erroneous values by recovering high frequency information; LSTM is used as it is suitable for complex time series data; various deep learning techniques, such as dilation convolution and attention, are used for extracting important information in the large datasets.

**Figure 6:**
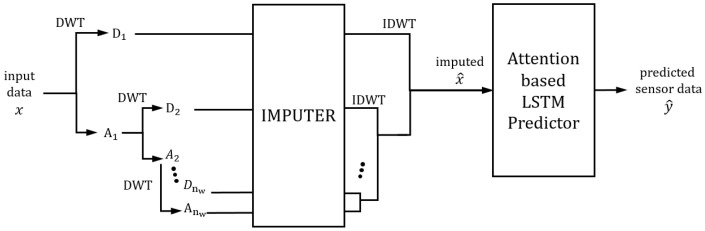
The architecture of the proposed network.

Network architectures are shown in Figure 6. The input data are decomposed by DWT to analyze the time series data effectively. The imputer network imputes the missing values for each decomposed part separately, as the data’s characteristics differ for the decomposed part per wavelet level and translation (approximated and detailed parts). The imputed decomposed data are inverse transformed by IDWT to make imputed data. The imputed data are then fed into the attention-based LSTM predictor. The attention mechanism is applied to both the feature and the temporal dimensions. The proposed structure is straightforward, as the imputer and predictor networks are based on the deep learning network. The details of each network are explained more closely below.

**Figure 7:**
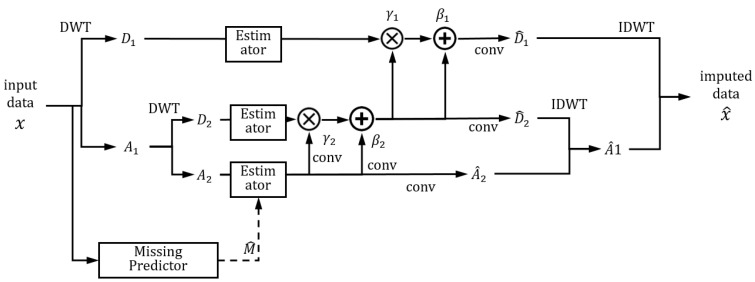
Details of the proposed imputer network.

The detailed structure of the imputer network is shown in Figure 7. It is effective to combine the proper analysis method with a deep learning network, and it can enhance performance. In visual odometry, the movement characteristics in images are effectively analyzed by optical flow, and the geometrical analysis combined with deep learning networks [42] improve performance. Similarly, the wavelet analysis was combined with a deep learning network in image inpainting and showed improved performance [21]. The proposed network also utilized the wavelet analysis method for analyzing the time series data effectively.

In the imputer network, the input data are decomposed ($D_{1},D_{2},A_{2}$) and estimated, respectively, as the characteristics of each decomposed part are different. The imputing network is regarded as a part of the generator in the GAN structure. For the discriminator, we constructed the discriminator for each decomposed part to reflect the different characteristics of each decomposed part. The missing mask is estimated ($\hat{M}$) using the input data, and the estimated mask assists the imputation. The convolution network is designed with dilation and gated convolution with residual techniques to use the information of both temporal and sensor dimensions. For imputing the detailed parts ($D_{1},D_{2}$), the data are adjusted by scaling (γ) and bias (β), as the detailed parts are sparse [21]. The imputed parts are inversely transformed (IDWT), and the imputed data ($\hat{x}$) is computed.

**Figure 8:**
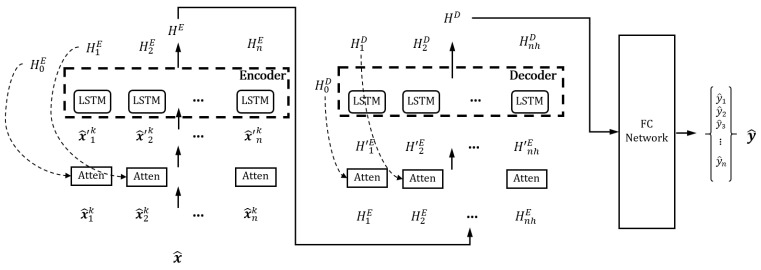
Details of the proposed attention-based LSTM predictor network.

The output of the imputer network is then input into the attention-based prediction network (Figure 8). The encoder–decoder structure of the prediction network can squeeze the abundant sensor information in the encoder and use the compressed context in the decoder for prediction. To predict the output sensor more accurately, the LSTM needs to concentrate on the important information in the imputed data ($\hat{x}$), and we utilized an attention mechanism. In the attention, the hidden state, cell state of the LSTM, and input data are used for selecting the important information adaptively [43]. The attention network includes the combination of fully connected layers, hyperbolic tangent, and softmax activation network to calculate attention weights, which are multiplied with the input to make attended input.

The attended input (${\hat{x}}^{\prime }$) is then fed into the encoder LSTM network, and the encoder calculates the cell state and hidden state. The decoder also uses the attention mechanism in the temporal dimension for the attended hidden state of the encoder (${H^{\prime }}^{E}$), and the hidden state of the decoder is calculated. Finally, the output of the decoder is fully connected with the concatenation of the encoder and decoder hidden state (the description is skipped in the figure for clarification) and outputs the estimated sensor data $\hat{y}$.

### 3.3. Loss Function

For the loss metric, the L1 norm is used, as it better learns the tendency. The MSE loss exaggerates the difference in the spiky noises, and the network learns the erroneous values. It also reproduces noise values and degrades the prediction of normal values. In the loss, the results in the process of the imputer network, the imputer network’s output, and the prediction network’s output are considered. The masks are estimated to distinguish whether the value is a missing value, which is used in the imputer network. The mask values are binary, and the mask loss is computed using the binary cross-entropy loss.

(11) $$L_{M}=-\frac{1}{CL}\sum_{i}^{C}\sum_{j}^{L}\left[M_{GT}^{ij}log\left({\hat{M}}^{ij}\right)+\left(1-M_{GT}^{ij}\right)log\left(1-{\hat{M}}^{ij}\right)\right]\;$$

where *C* is the number of the input channel and *L* is the length of the data.

We take into account the estimation of the decomposed parts through wavelet loss. The wavelet loss ($L_{W}$) considers the imputed approximated part and the detailed parts. The imputed approximated part ($\hat{A}$) and the imputed detailed parts (${\hat{D}}_{n}$) are compared with true values through the L1 norm as below.

(12) $$L_{W}=\frac{2^{n_{w}}}{CL}\left|\right|\hat{A}-A_{GT}{\left|\right|}_{1}+\sum_{n=1}^{n_{w}}\frac{2^{n}}{CL}\left|\right|{\hat{D}}_{n}-D_{n_{GT}}{\left|\right|}_{1}\;$$

where $n_{w}$ denotes the maximum number of wavelet decomposition levels.

To ensure the imputer network correctly recovered the input value, we consider imputed loss ($L_{I}$) by comparing the imputed value ($\hat{x}$) with true input ($x_{GT}$). Similarly, the final prediction output is considered through the regression loss ($L_{R}$) using the L1 norm of the predicted ($\hat{y}$) and true sensor output ($y_{GT}$).

(13) $$L_{I}=\frac{1}{CL}\left|\right|\hat{x}-x_{GT}{\left|\right|}_{1}$$

(14) $$L_{R}=\frac{1}{L}\left|\right|\hat{y}-y_{GT}{\left|\right|}_{1}$$

We adopt WGAN-GP for better performance and higher training stability, and the discriminator loss function follows the original paper [30]. We set discriminators for each decomposed part, and the discriminator loss is the summation of the loss of each approximated and detailed part. In training, we trained the discriminator five times per the one training of the generator, as specified in the paper. For each decomposed part, the discriminator is constructed, and the discriminator loss ($L_{D}$) and the adversarial ($L_{Adv}$) loss is formulated as below.

(15) $$L_{Adv}=-E\left[D_{A}\left(\hat{A}\right)\right]-\sum_{n=1}^{n_{w}}E\left[D_{D_{n}}\left({\hat{D}}_{n}\right)\right]$$

(16) $$\begin{array}{c} L_{D}=E\left[D_{A}\left(A_{GT}\right)\right]\;-E\left[D_{A}\left(\hat{A}\right)\right]+\lambda E[(||\nabla D_{A}\left(\tilde{A}\right){\left||-1\right)}^{2}]+\;\\ \sum_{n=1}^{n_{w}}[E\left[D_{D_{n}}\left(D_{n_{GT}}\right)\right]-E\left[D_{D_{n}}\left({\hat{D}}_{n}\right)\right]+\lambda E[(||\nabla D_{D_{n}}\left({\tilde{D}}_{n}\right){\left||-1\right)}^{2}]] \end{array}$$

where D is the discriminator of each decomposed part with the subscript *D* and *A* meaning detailed and approximated, E[·] implies expectation, ${\tilde{D}}_{n}=\varepsilon D_{n_{GT}}+\left(1-\varepsilon \right)\hat{D_{n}}$, $\tilde{A}=\varepsilon A_{GT}+\left(1-\varepsilon \right)\hat{A}$ with ε∼U[0,1], and λ is set to ten.

In the proposed network, the generator can be regarded as the whole network, including the imputer network and the prediction network. Therefore, the generator loss is expanded to the addition of the adversarial loss and the other losses with the balancing terms. In the test, the balancing terms were set to one as the losses were normalized with the data size. The full objective is the maximization of the discriminator loss and the minimization of the generator loss.

(17) $$L_{G}=L_{Adv}+\lambda_{M}L_{M}+\lambda_{W}L_{W}+\lambda_{I}L_{I}+\lambda_{R}L_{R}$$

## 4. Test Result

### 4.1. Test Setup

**Figure 9:**
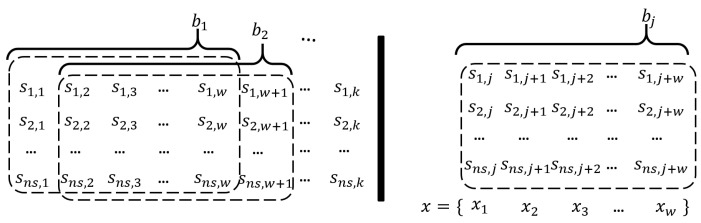
Preparation of input data.

The proposed algorithm is trained and tested with five sets of flight data. Each flight dataset is unique, as the flight tests are conducted in different settings and environments (trajectories, speed, control, etc.). Therefore, the datasets for the training and the testing should be separated. In the five flight datasets, we used four datasets in training and one in the test. In preparation for training, the batches are generated through a sliding window and multiple batches are trained simultaneously. In the flight test dataset, if there exist ns sensors and *k* data length, batches are generated through the sliding window process, as shown in the left part of the Figure 9. In each batch, the *x* are inputted to the network sequentially from $x_{1}$ to $x_{w}$, as in the right part of the Figure 9 ($b_{j}$ represents an arbitrary batch). In total, we used 230 of sensor data and about 130k data length.

All the compared methods used the same batch size and window size of the data for the preparation of the input. Parameters related to the training and testing are stated in Table 3. For the learning rate, inverse sqrt scheduling is used.

Generally, the categories of missing values are divided as missing completely at random (MCAR), missing at random (MAR), and missing not at random (MNAR). In the missile dataset, missing values can occur in communication loss, sensor error, and random error, as explained below. The probability of missing is completely random in these cases, and we assumed the missing values as MCAR.

**Figure 10:**
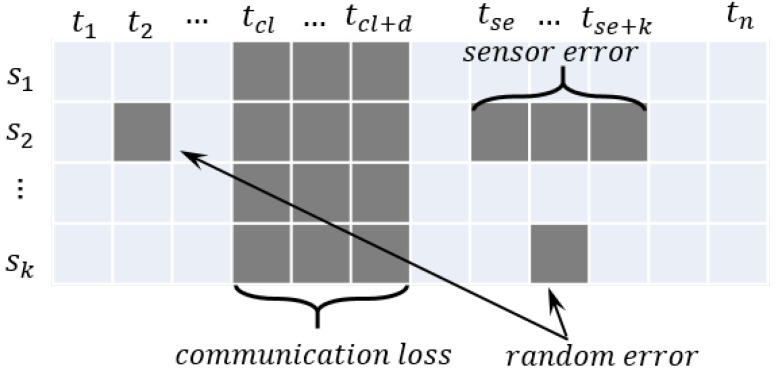
Examples of missing values cases in the dataset.

In the flight data, the causes of missing can be divided into three cases as below, and the three cases are explained in Figure 10.

1. Communication loss: Communication loss can occur at a random time for random time intervals, and all the data are lost. The missing values are generated at a random time (cl) for random time intervals (*f*).
2. Sensor error: Sensor error can be caused by contact problems or corruption of sensors, and the specific sensor data are missing. The missing values are generated for the random sensor data for a random time (se) for random time intervals (*k*).
3. Random error: Random error can be caused by random noise or an unknown reason, and the missing values are generated for a random feature and random time.

Most of the missing values in the three cases are caused by communication loss, and we only considered the first case in this paper. The missingness is defined as the missing rate, which is the percentage of the number of missing values over the number of normal values. The missing values are generated completely randomly, and the missing rate varies slightly between the missing generations, but we marked the average value, as the difference is very small and negligible. In the test, we tested with five missing rates for the performance evaluation.

To evaluate the prediction performance, we compared the results with the four commonly used statistical metrics, e.g., mean absolute error (MAE), mean squared error (MSE), root mean squared error (RMSE), and mean absolute percentage error (MAPE).

### 4.2. Performance Evaluation

For the performance evaluation, the proposed network is compared with three different deep learning prediction models. In the different models, the basic LSTM model consists of multiple dilations, fully connected layers, and LSTM networks. Wavelet analysis with LSTM (WLSTM) consists of wavelet reconstruction method, dilation, and LSTM networks. Attention LSTM (ALSTM) is an encoder–decoder LSTM with attention techniques. The proposed method combined the wavelet method with attention LSTM with normalization and GAN architecture to reconstruct the detailed information of the wavelet decomposed data. For a fair comparison, we set the same network parameters for the LSTM used in all models of the tests, such as the number of layers and the number of hidden layers.

#### 4.2.1. Quantitative Evaluation

First, we tested the networks with clean data (without missing values). Some sensors can be predicted relatively easily due to simple statistical characteristics, and some cannot. Therefore, the networks were tested for various sensors, and the test results are the averaged values of several test results from different sensors. The comparison result (Table 4) shows that the proposed method predicts the sensor most accurately for all four metrics. Basic LSTM shows inferior results compared to the other models, because it fails to predict some sensor values. It results in the model having large variations in metrics, but the proposed method shows very stable predictability in all sensors. The results of basic LSTM are omitted in the subsequent results, as it fails to predict the sensor value, so the comparison is meaningless.

Second, we tested the methods with the five different missing rates (Table 5). In comparison, MAE, MSE, and RMSE metrics were used. It is obvious that as the missing rate increases, the prediction performance degrades. When the missing rate is low, the performance is degraded slightly because the prediction performance is only slightly affected. In the resulting table, some metrics show similar results when the missing rate is low, and the differences are shown more clearly when the missing rate is high. In all cases, the proposed method shows the best results for all the missing rates.

#### 4.2.2. Qualitative Evaluation

**Figure 11:**
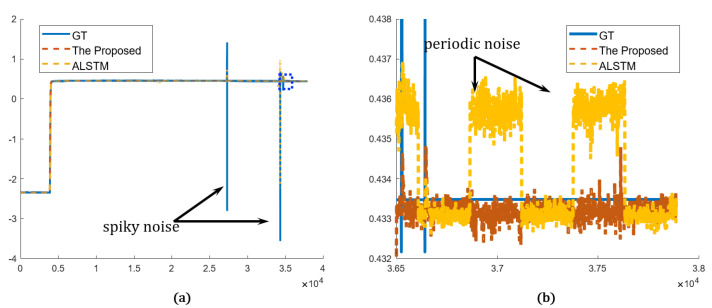
Prediction results in time domain (sensor A): (**a**) overview of the proposed and the best in other methods compared to the GT; (**b**) detailed view of the blue dotted rectangle of (**a**).

**Figure 12:**
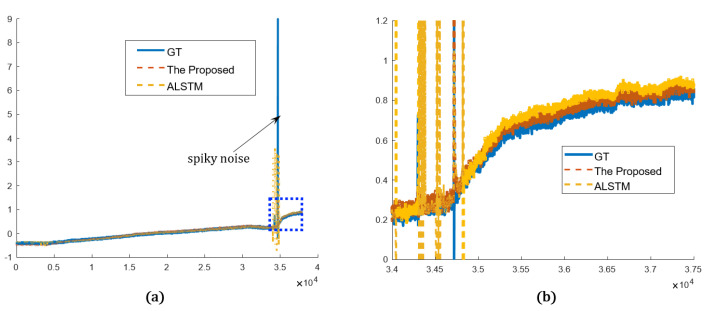
Prediction results in time domain (sensor B): (**a**) overview of the proposed and the best of other methods compared to the GT; (**b**) detailed view of the blue dotted rectangle of (**a**).

The proposed network is compared in both the time and frequency domains in the qualitative evaluation. The graphs plot only the proposed method and the best results of other methods for simple comparison. The overview of the prediction results shows that both the proposed and other methods predict the sensor values very close to the real sensor value, ground truth (GT). Both methods predict abrupt and gradual changes relatively well. In addition, in the prediction results, the spiky noise is less than that of the GT, and the spiky noises are alleviated. In the magnified view, however, the proposed network predicted the sensor more accurately and stably than the best of the others (ALSTM). In Figure 11, periodic noises are included in the result from ALSTM, which were caused by the spiky noises. On the other hand, the proposed method is less affected by the spiky noises, as it analyzes data using wavelet transform, so it predicted the sensor data robustly. Sensor A and sensor B represent the arbitrary sensors. In Figure 12, GT shows more repeated spiky noises compared to Figure 11. ALSTM is affected by excessive spiky noises, and, consequently, many spiky noises are generated. However, the proposed predictor follows the tendency of the GT, and the spiky noises are rejected by utilizing the wavelet analysis.

**Figure 13:**
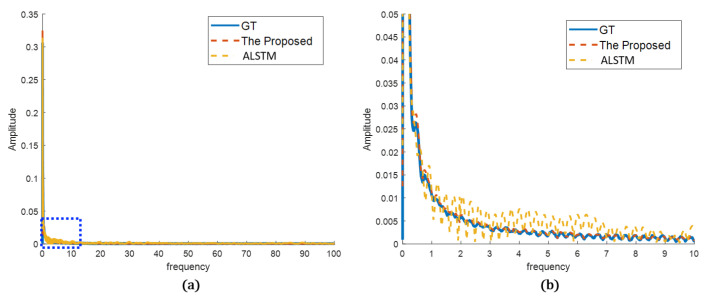
Prediction results in frequency domain (sensor A): (**a**) overview of the proposed and the best of the other methods compared to the GT; (**b**) detailed view of the blue dotted rectangle of (**a**).

**Figure 14:**
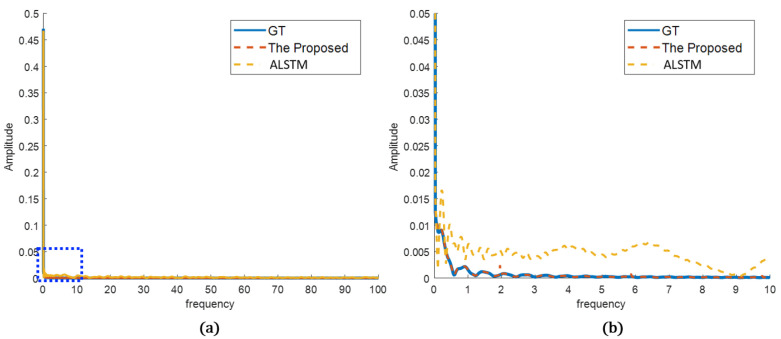
Prediction results in frequency domain (sensor B): (**a**) overview of the proposed and the best of the other methods compared to the GT; (**b**) detailed view of the blue dotted rectangle of (**a**).

In the frequency analysis, the prediction results were transformed by Fourier transform to compare the frequency characteristics of the sensor data. As can be seen, the differences are more clear. In Figure 13, ALSTM follows the frequency tendency of the GT, but it has oscillations or errors compared to the GT. In contrast, the proposed method shows frequency characteristics very close to the GT. It better predicted the true sensor in both frequency tendency and characteristics. In Figure 14, both the proposed method and ALSTM seem to predict the frequency characteristics well. However, the magnified view shows that the ALSTM predicts high frequencies much larger than the GT, and it is caused by the overproduced spiky noises. The proposed method suppresses the spiky noises in the prediction, and the frequency characteristics are very close to the GT, even in the magnified view.

As noted above, the proposed method utilizes wavelet analysis and GAN architecture. The wavelet analysis interprets frequency information more precisely, and GAN architecture can generate detailed information in competing between the generator and the discriminator. It results in the predicted sensor data being closer to the true sensor values.

## 5. Conclusions

This paper proposes a deep learning-based prediction network for sensor data from missile flight tests. The missile datasets are highly different and independent from test to test, as environment and planning differ for each flight. Further, data are incomplete due to communication errors or loss, different sampling rates/time standards, and the number of sensor data is over two hundred with complex correlations. Therefore, the incompleteness, complexity, and independencies among datasets make it difficult to predict the data.

The proposed network utilizes wavelet decomposition in analyzing the sensor data and reconstructs the incomplete data using the recent GAN architectures with better training stability and performance. The interpretability of time series data with wavelet analysis and the advantage of GAN helps to handle incomplete and complex data accurately. The final outputs are predicted by focusing on the important information using the LSTM network with an attention mechanism. The proposed network combined appropriate network structures considering the wavelet technique suitable for analyzing temporal data and the characteristics of the state-of-the-art deep learning technology, and the new network structure can predict sensors accurately.

The test result shows that the proposed network can effectively predict the missile sensor data in quantitative and qualitative aspects. The accuracy and stability of the proposed network outperform the previous methods in the MAE, MSE, RMSE, and MAPE metrics. The qualitative evaluation showed that the proposed network predicted the target sensor well in statistical and frequency analysis. The proposed network can be applied to reproduce corrupted or broken sensors in flight tests when unexpected situations occur.

## Figures and Tables

**Table 1:** Categories of sensor prediction methods.

		Advantage	Disadvantage
Test-based modelling		Computationally compact	Time consuming in analyzing and modeling Needs multiple tests in a limited environment setting
Data driven	Traditional (ARMA, ARIMA)	Can predict data after the test	Difficulty in achieving high performance in a complex system Need expert’s preprocessing
	Deep learning -based method	Effective and accurate with little human effort Various methods can be combined in the network	Requires computing power for training the large model

**Table 2:** An example of missile data (sensor and intra-communication).

Time	Data1	Data2	Data3	Data4	Data5	Data6	Data7	Data8	Data9	Data10	Data11
50.433 s	0.9940	2.7940	0.9830	10,070	−1.423	827.42	23.816	22.346	NaN	41.806	24.032
50.437 s	NaN	NaN	NaN	NaN	NaN	NaN	NaN	NaN	45.2868	NaN	NaN
50.438 s	0.9940	2.7940	0.9830	10,070	−1.423	827.42	23.780	23.346	NaN	41.792	24
50.442 s	NaN	NaN	NaN	NaN	NaN	NaN	NaN	NaN	45.2868	NaN	NaN
50.443 s	0.9940	2.7940	0.9830	10,070	−1.423	827.42	23.780	22.325	NaN	41.792	24
50.447 s	NaN	NaN	NaN	NaN	NaN	NaN	NaN	NaN	45.0913	NaN	NaN
50.448 s	0.9940	2.7940	0.9830	10,070	−1.423	827.42	23.754	22.325	NaN	41.758	23.983
50.452 s	NaN	NaN	NaN	NaN	NaN	NaN	NaN	NaN	45.2868	NaN	NaN
50.453 s	0.9940	2.7930	0.9830	10,070	−1.423	827.42	23.754	22.308	NaN	41.758	23.983
50.457 s	NaN	NaN	NaN	NaN	NaN	NaN	NaN	NaN	45.2868	NaN	NaN
50.458 s	0.9940	2.7930	0.9830	10,070	−1.423	827.42	23.733	22.308	NaN	41.734	23.974
50.462 s	NaN	NaN	NaN	NaN	NaN	NaN	NaN	NaN	45.6778	NaN	NaN
50.463 s	1	2.7930	0.9830	10,070	−1.423	826.91	23.733	22.284	NaN	41.734	23.974
50.467 s	NaN	NaN	NaN	NaN	NaN	NaN	NaN	NaN	45.0913	NaN	NaN
50.468 s	1	2.7930	0.9830	10,082	−1.423	826.91	23.716	22.284	NaN	41.711	23.923

**Table 3:** Parameters in training and testing.

Model	Parameters
No. iterations	200
Learning rate	0.001
Optimizer	Adam optimizer
Batch size	512
Window size	128
No. LSTM layer	2
No. hidden layer	64
Dropout in LSTM	0.4

**Table 4:** Quantitative comparison without missing data.

Model	MAE	MSE	RMSE	MAPE
Basic LSTM	0.454	0.555	0.676	5.184
WLSTM	0.075	0.022	0.126	1.290
ALSTM	0.041	0.073	0.215	0.951
Proposed Method	0.035	0.014	0.077	0.489

**Table 5:** Quantitative comparison with different missing rates. (The top, middle, and bottom represent MAE, MSE, and RMSE values, respectively).

Model	Missing Rate				
	1.15%	2.3%	4.6%	9%	17%
WLSTM	0.067	0.071	0.066	0.081	0.099
	0.022	0.023	0.023	0.029	0.041
	0.127	0.133	0.128	0.147	0.179
ALSTM	0.045	0.049	0.056	0.070	0.097
	0.081	0.078	0.071	0.081	0.088
	0.226	0.227	0.227	0.241	0.263
Proposed Method	0.037	0.039	0.044	0.053	0.070
	0.016	0.017	0.021	0.025	0.039
	0.091	0.097	0.115	0.129	0.163

## Data Availability

Not applicable.

## References

[1] Leon-Medina J.X., Camacho J., Gutierrez-Osorio C., Salomón J.E., Rueda B., Vargas W., Sofrony J., Restrepo-Calle F., Tibaduiza D.T. (2021). Temperature Prediction Using Multivariate Time Series Deep Learning in the Lining of an Electric Arc Furnace for Ferronickel Production. Sensors.

[2] Macias E., Boquet G., Serrano J., Vicario J.L., Ibeas J., Morel A. Novel imputing method and deep learning techniques for early prediction of sepsis in intensive care units. Proceedings of the 2019 Computing in Cardiology.

[3] Nikparvar B., Rahman M., Hatami F., Thill J.C. (2021). Spatio-temporal prediction of the COVID-19 pandemic in US counties: Modeling with a deep LSTM neural network. Sci. Rep..

[4] Han L., Yu C., Xiao K., Zhao X. (2019). A new method of mixed gas identification based on a convolutional neural network for time series classification. Sensors.

[5] Li Y., Zhu Z., Kong D., Han H., Zhao Y. (2019). EA-LSTM: Evolutionary attention-based LSTM for time series prediction. Knowl.-Based Syst..

[6] Rhif M., Ben Abbes A., Farah I.R., Martínez B., Sang Y. (2019). Wavelet transform application for/in non-stationary time-series analysis: A review. Appl. Sci..

[7] Kim T., Ko W., Kim J. (2019). Analysis and Impact Evaluation of Missing Data Imputation in Day-ahead PV Generation Forecasting. Appl. Sci..

[8] Fu Y., He H.S., Hawbaker T.J., Henne P.D., Zhu Z., Larsen D.R. (2019). Evaluating k-Nearest Neighbor (k NN) Imputation Models for Species-Level Aboveground Forest Biomass Mapping in Northeast China. Remote Sens..

[9] Valdiviezo H.C., Van Aelst S. (2015). Tree-based prediction on incomplete data using imputation or surrogate decisions. J. Inf. Sci..

[10] Zhang X., Yan C., Gao C., Malin B., Chen Y. XGBoost imputation for time series data. Proceedings of the 2019 IEEE International Conference on Healthcare Informatics.

[11] Krithiga R., Ilavarasan E. (2021). Hyperparameter tuning of AdaBoost algorithm for social spammer identification. Int. J. Pervasive Comput. Commun..

[12] Mikhchi A., Honarvar M., Kashan N.E.J., Zerehdaran S., Aminafshar M. (2017). Analyses and comparison of K-nearest neighbour and AdaBoost algorithms for genotype imputation. Res. Vet. Sci..

[13] Lall R., Robinson T. (2022). The MIDAS touch: Accurate and scalable missing-data imputation with deep learning. Political Anal..

[14] Zhuang Y., Ke R., Wang Y. (2019). Innovative method for traffic data imputation based on convolutional neural network. IET Intell. Transp. Syst..

[15] Cao W., Wang D., Li J., Zhou H., Li L., Li Y. Brits: Bidirectional recurrent imputation for time series. Proceedings of the 32nd Conference on Neural Information Processing Systems (NeurIPS 2018).

[16] Yoon J., Jordon J., Schaar M. Gain: Missing data imputation using generative adversarial nets. Proceedings of the 35th International Conference on Machine Learning.

[17] Nibhanupudi S., Youssif R., Purdy C. Data-specific signal denoising using wavelets, with applications to ECG data. Proceedings of the 2004 47th Midwest Symposium on Circuits and Systems.

[18] Joo T.W., Kim S.B. (2015). Time series forecasting based on wavelet filtering. Expert Syst. Appl..

[19] Ryu S., Koo G., Kim S. W. (2020). An Adaptive Selection of Filter Parameters: Defect Detection in Steel Image Using Wavelet Reconstruction Method. ISIJ Int..

[20] Memarian S. O., Asgari J., Amiri-Simkooei A. (2022). Wavelet decomposition and deep learning of altimetry waveform retracking for Lake Urmia water level survey. Mar. Georesources Geotechnol..

[21] Yu Y., Zhan F., Lu S., Pan J., Ma F., Xie X., Miao C. Wavefill: A wavelet-based generation network for image inpainting. Proceedings of the IEEE/CVF International Conference on Computer Vision.

[22] Guo T., Seyed Mousavi H., Huu Vu T., Monga V. Deep wavelet prediction for image super-resolution. Proceedings of the IEEE Conference on Computer Vision and Pattern Recognition Workshops.

[23] Sarhan A.M. (2020). Brain tumor classification in magnetic resonance images using deep learning and wavelet transform. J. Biomed. Eng..

[24] Goodfellow I., Pouget-Abadie J., Mirza M., Xu B., Warde-Farley D., Ozair S., Aaron C., Bengio Y. (2014). Generative adversarial nets. Advances in neural information processing systems. Adv. Neural Inf. Process. Syst..

[25] Xian W., Sangkloy P., Agrawal V., Raj A., Lu J., Fang C., Yu F., Hays J. Texturegan: Controlling deep image synthesis with texture patches. Proceedings of the IEEE Conference on Computer Vision and Pattern Recognition.

[26] Demir U., Unal G. Patch-based image inpainting with generative adversarial networks. Proceedings of the Conference on Computer Vision and Pattern Recognition.

[27] Zhang W., Li X., Jia X.D., Ma H., Luo Z., Li X. (2020). Machinery fault diagnosis with imbalanced data using deep generative adversarial networks. Measurement.

[28] Arjovsky M., Chintala S., Bottou L. Wasserstein generative adversarial networks. Proceedings of the International Conference on Machine Learning.

[29] Saxena D., Cao J. (2021). Generative adversarial networks (GANs) challenges, solutions, and future directions. ACM Comput. Surv..

[30] Gulrajani I., Ahmed F., Arjovsky M., Dumoulin V., Courville A.C. (2017). Improved training of wasserstein gans. Adv. Neural Inf. Process. Syst..

[31] Connor J.T., Martin R.D., Atlas L.E. (1994). Recurrent neural networks and robust time series prediction. IEEE Trans. Neural Netw. Learn. Syst..

[32] Hochreiter S., Schmidhuber J. (1997). Long short-term memory. Neural Comput..

[33] Tiwari G., Sharma A., Sahotra A., Kapoor R. English-Hindi neural machine translation-LSTM seq2seq and ConvS2S. Proceedings of the 2020 International Conference on Communication and Signal Processing.

[34] Karim F., Majumdar S., Darabi H., Harford S. (2019). Multivariate LSTM-FCNs for time series classification. Neural Netw..

[35] Liu X., Lin Z., Feng Z. (2021). Short-term offshore wind speed forecast by seasonal ARIMA-A comparison against GRU and LSTM. Energy.

[36] Yamak P.T., Yujian L., Gadosey P.K. A comparison between arima, lstm, and gru for time series forecasting. Proceedings of the 2019 2nd International Conference on Algorithms, Computing and Artificial Intelligence.

[37] Chung J., Gulcehre C., Cho K., Bengio Y. (2014). Empirical evaluation of gated recurrent neural networks on sequence modeling. Neural and Evol. Comput..

[38] Shewalkar A. (2019). Performance evaluation of deep neural networks applied to speech recognition: RNN, LSTM and GRU. J. Artif. Intell. Soft Comput. Res..

[39] Shewalkar A.N. (2018). Comparison of rnn, lstm and gru on speech recognition data. Masters Thesis.

[40] Yang S., Yu X., Zhou Y. Lstm and gru neural network performance comparison study: Taking yelp review dataset as an example. Proceedings of the 2020 International Workshop on Electronic Communication and Artificial Intelligence.

[41] Zarzycki K., Ławryńczuk M. (2021). LSTM and GRU neural networks as models of dynamical processes used in predictive control: A comparison of models developed for two chemical reactors. Sensors.

[42] Zhan H., Weerasekera C.S., Bian J.W., Reid I. Visual odometry revisited: What should be learnt?. Proceedings of the 2020 IEEE International Conference on Robotics and Automation.

[43] Qin Y., Song D., Chen H., Cheng W., Jiang G., Cottrell G. (2017). A dual-stage attention-based recurrent neural network for time series prediction. Mach. Learn..

